# Dipping Technique for Ureteroileal Anastomosis in Orthotopic Ileal Neobladder: 20-Year Experience in 670 Patients—No Stenosis with Preservation of the Upper Tract

**DOI:** 10.1155/2013/725286

**Published:** 2013-05-26

**Authors:** Mohamed M. Wishahi, Hosam Elganzoury, Amr Elkhouly, Ahmed Mehena

**Affiliations:** Urology Department, Theodor Bilharz Research Institute, Cairo, Egypt

## Abstract

*Objectives*. Many techniques were described for ureteroileal anastomosis in orthotopic bladder substitution, ranging from nonrefluxing to refluxing techniques, all aiming at preservation of the upper tract. We describe our technique of dipping the ureter into the ileal pouch, which is simple and had no complications. *Patients and Methods*. Our technique implies dipping the ureter in the lateral side of the pouch, in right and left corners, with two rows of four sutures fixing the seromuscular layer of the ureter to the seromuscular layer of the ileal pouch. The procedure was applied in both normal ureteric calibre and dilated ureter. Total number of procedures done was 1,340 ureters in 670 patients after radical cystectomy for invasive carcinoma of the bladder of urothelial and nonurothelial cancer. *Results*. Followup of patients every six months and onward did not show stenosis in the ureteroileal anastomotic site. Filling of the ureter with contrast dye on ascending pouchogram was observed in patients who had considerably dilated ureters at the time of cystectomy. Normal ureter did not show clinical reflux but radiological filling of the ureter when the intravesical pressure exceeded the leak point pressure. Time to perform the dipping technique was 5–7 minutes for each site. *Conclusion*. Dipping technique for ureteroileal anastomosis in orthotopic ileal neobladder avoids the incidence of stenosis, preserves the upper tract, is a fast procedure, stands the evaluation in long-term followup, and was practiced successfully for twenty years.

## 1. Introduction

Since the introduction of orthotopic ileal neobladder in 1985, the procedure gained wide acceptance and has been practiced by most urologist. The turning point in the technique of orthotopic ileal neobladder was the introduction of the detubularised ileal pouch that ensured low pressure reservoir protecting the upper tract from high-pressure reflux. Many techniques addressed the antireflux procedures in the ureteroileal anastomosis (UIA), which were first described by Le duc et al. [1], simultaneously Koch described the antireflux intussuscepted nipple that was widely applied [2]; Studer et al. introduced the technique of UIA to an afferent tubular isoperistaltic segment [3, 4]; Abol-Enein et al. introduced the procedure of serous-lined extramural tunnel which was described for normal and dilated ureter [5, 6]. The era of antireflux UIA was followed by the introduction of the concept reflux or nonreflux ureteric anastomosis [7, 8]. There were and still are arguments on performing which technique. Stenosis had been reported with varying incidence in most series; the incidence of reflux detected by pouchogram was reported in most series.

## 2. Materials and Patients

From 1993 to 2013, we applied the dipping technique in the ureteroileal anastomosis in orthotopic detubularised ileal neobladder substitution in 670 patients after radical cystectomy (RC) for invasive carcimoma of the bladder. 560 were men with age ranging from 45 to 75 (mean 57 ∓ 5 years) and 110 women with age range 35–65 (mean 52 ± 4 years). Invasive urothelial carcinoma was in 330 patients while squamous cell carcinoma was in 340 patients. The dipping technique was performed in the 670 patients on both sides for UIA with a total of 1340 procedures. The dipping technique was applied in normal calibre ureters and in dilated ureters with varying degree of dilatation. Followup after RC and orthotopic ileal neobladder was every 6 months and onward in order to assess kidney function and to monitor changes and dilatation of the upper tract. It was done by ultrasonography, intravenous urography, and ascending pouchogram being done simultaneously with urodynamic recording of the intrapouch pressure and the leak point.

## 3. Technique

After RC for invasive carcinoma of the bladder, an isolated ileal segment of 35 cm is selected and detubularised, a pouch is constructed in a spherical or U-shape, and then it is closed except for the 2 cm width opening in its lower part which will receive the urethraileal anastomosis.

**Figure 1:**
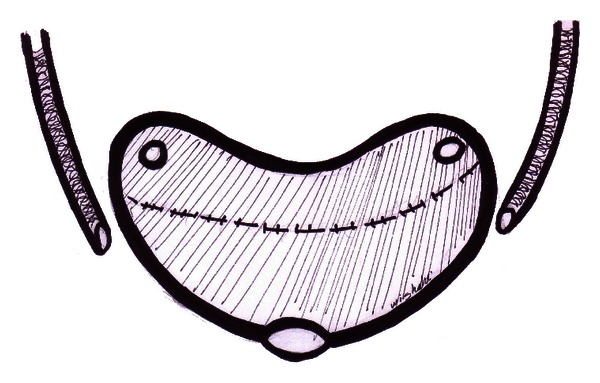
Dipping technique: construction of the detubularized ileal pouch U-form with complete closure apart from the site of urethral anastomosis, two holes being made in the lateral sided of the pouch to receive the two ureters.

**Figure 2:**
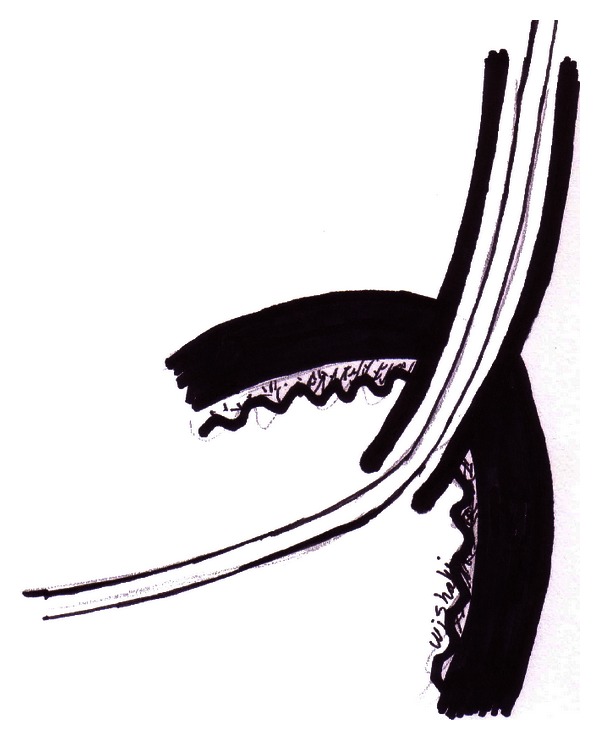
Dipping technique: the ureter passed the pouch hiatus, and the drainage ureteric stent passes out of the pouch from a separate stab opening.

On either side of the pouch in its lateral aspect, a hole is opened with a diameter equal to the ureteric calibre; the hole is done using a scalpel with haemostasis using bipolar coagulation for the submucosal small vessels in the ileal wall (Figure 1). An 80 cm drainage ureteric stent is passed into the kidney through the opened ureteric end and anchored to the ureteric end with 0/5 polyglycolic acid sutures; the ureteric drainage tube is passed through the pouch hole and gets out of the pouch from a lower positioned stab using an 8 Fr metal probe (Figure 2). 

**Figure 3:**
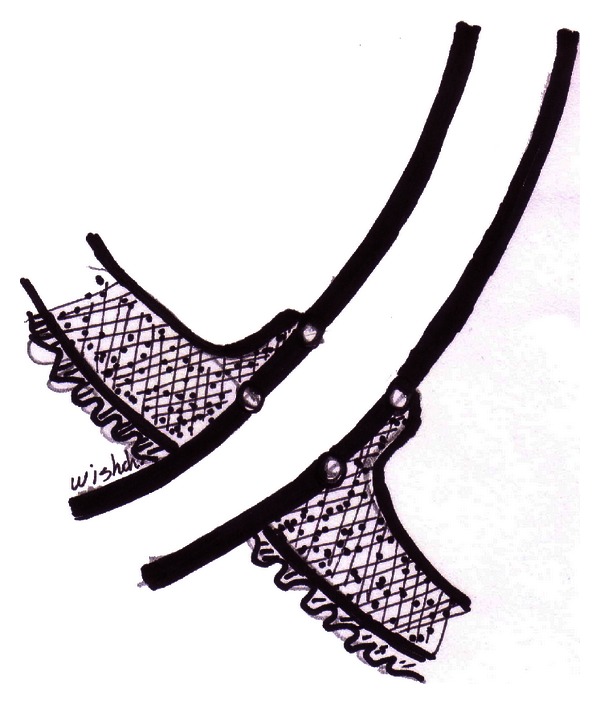
Dipping technique: the anchoring of the ureter to the ileal wall of the ileal pouch with two rows of sutures.

The ureteral end is introduced into the pouch hole for a length of 1 cm to allow secure anastomosis; the ileal pouch wall of the seromuscular layer is anchored to the serosa muscularis of the outer wall of the ureter; sutures are arranged in two rows each with four sutures with 0/4 polyglycolic acid sutures (Figure 3).

**Figure 4:**
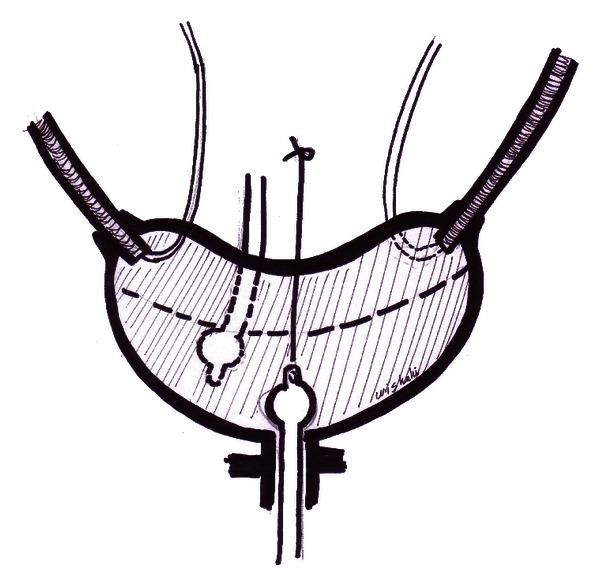
Dipping technique. the final pouch anastomosis to the urethra, pouch; and kidney drainage achieved with balloon uretheral catheter refixed with Harris stitch, suprapubic tube, and two drainage ureteric stents.

Final step is the anastomosis of the pouch to the urethral stump with four 0/3 sutures; the pouch is drained with a ballon silicone catheter anchored to the abdominal wall with Harris suture and with a supra pubic pouch catheter, two kidney drainage catheters, and one pelvic drainage tube (Figure 4). The operative time for the dipping technique for one UIA is 7 minutes. The technique was applied to normal calibre or dilated ureter. Ascending pouchogram was done in association with urodynamic pouch pressure measurement and measurement of the leak point pressure.

## 4. Results

**Figure 5:**
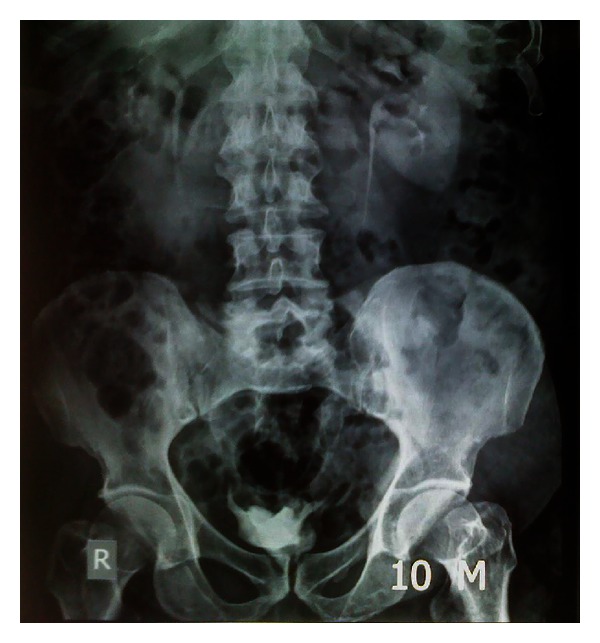
Five years postoperative urogram showing perfect upper tract with no stenosis, preservation of the kidneys, and normal caliber of the ureters.

Followup of the 670 patients, every 6 months and onward, was done with kidney function tests, urine analysis, and imaging. Stenosis was not detected in all the 1,340 ureteroileal anastomosis using the dipping technique (Figure 5); radiological filling of the ureter with contrast solution in pouchogram was detected in 15% of ureters being dilated prior to RC; in normal ureter the incidence of ureteral filling was 25% and was unilateral. Ureteral filling was detected in urodynamic pouchogram when the pouch pressure was over 35 cm H_2_O; the resting pouch pressure was 15–25 cm H_2_O. Patients with the radiological finding of ureteral filling did not have severe urinary tract infection or febrile attacks. Deterioration of the upper tract was detected in 10 patients who developed urethral stricture that was treated with visual internal urethrotomy and pouch drainage for two weeks.

## 5. Discussion

There are numerous published series on the need for an antireflux technique for UIA [1–6], and there are also many publications addressing the reflux technique. The incidence of stenosis in the UIA varies in different series from 0% to 20% with consequent upper tract deterioration. The dipping technique applied in 1,340 renal units in orthotopic ileal neobladder substitution aimed at a nontraumatising, anatomical, simple technique that avoids mucosa to mucosa anstomosis of the ureteric wall to the ileal wall, by this way avoiding the incidence of stenosis. The technique implies seromuscolar sutures between the two walls of the ureter and the ileum.

The detubularized pouch in both shapes, spherical or U-shape, ensures a low pressure reservoir with a pouch pressure lower than 30 cm H_2_O. Further elevation of intrapouch pressure will reach the leak point which will protect the upper tract from high pressure reflux. Low pressure reflux was observed to have no influence on the upper tract. The finding of ureteral filling during ascending pouchogram should not be considered as reflux, but should be labelled as imaging finding without adverse effects. 

## 6. Conclusion

Dipping technique for ureteroileal anastomosis in ileal neobladder substitution proved to be without complications, and preserved the upper tract from the hazards of stenosis. The radiological finding of ureteral filling in pouchogram was associated with high pressure pouch during artificial filling. In nonartificial situations, this ureteral filling happens in case of high intrapouch pressure as a result of uretheral strictures.
